# The long-term effects of maternal deprivation on the number and size of inhibitory interneurons in the rat amygdala and nucleus accumbens

**DOI:** 10.3389/fnins.2023.1187758

**Published:** 2023-06-26

**Authors:** Dubravka Aleksic, Joko Poleksic, Gorana Agatonovic, Vuk Djulejic, Maja Vulovic, Miljana Aksic, Gebhard Reiss, Mohammad I. K. Hamad, Igor Jakovcevski, Milan Aksic

**Affiliations:** ^1^School of Medicine, Institute of Anatomy “Niko Miljanić”, University of Belgrade, Belgrade, Serbia; ^2^Department of Anatomy, Faculty of Medical Sciences, University of Kragujevac, Kragujevac, Serbia; ^3^Center for Medical Biochemistry, University Clinical Center of Serbia, Belgrade, Serbia; ^4^Institut für Anatomie und Klinische Morphologie, Universität Witten/Herdecke, Witten, Germany; ^5^Department of Anatomy, College of Medicine and Health Sciences, United Arab Emirates University, Al Ain, United Arab Emirates

**Keywords:** amygdala, GABAergic interneurons, nucleus accumbens, maternal deprivation, parvalbumin

## Abstract

**Introduction:**

There is an increasing evidence supporting the hypothesis that traumatic experiences during early developmental periods might be associated with psychopathology later in life. Maternal deprivation (MD) in rodents has been proposed as an animal model for certain aspects of neuropsychiatric disorders.

**Methods:**

To determine whether early-life stress leads to changes in GABAergic, inhibitory interneurons in the limbic system structures, specifically the amygdala and nucleus accumbens, 9-day-old Wistar rats were exposed to a 24 h MD. On postnatal day 60 (P60), the rats were sacrificed for morphometric analysis and their brains were compared to the control group.

**Results:**

Results show that MD affect GABAergic interneurons, leading to the decrease in density and size of the calcium-binding proteins parvalbumin-, calbindin-, and calretinin-expressing interneurons in the amygdala and nucleus accumbens.

**Discussion:**

This study indicates that early stress in life leads to changes in the number and morphology of the GABAergic, inhibitory interneurons in the amygdala and nucleus accumbens, most probably due to the loss of neurons during postnatal development and it further contributes to understanding the effects of maternal deprivation on brain development.

## Introduction

Maternal deprivation (MD) is a widely used paradigm for the investigation of neurobiological changes associated with vulnerability to stress-related diseases in animal models. This model consists of the separation of newborn infants from their mothers for 24 h, on the 9th day after birth, and during this period they are not fed by their mothers (Llorente et al., 2007). The separation of pups from their mothers increases plasma corticosterone, which causes disturbances in postnatal development (Llorente et al., 2007). Namely, maternal deprivation reduces prepulse inhibition and latent inhibition, and it enchances sensitivity to dopaminergic drugs (Ellenbroek et al., 2005). Numerous studies show that changes in different brain structures and changes in behavior later in life are not diminished when the stressor is removed. Early life stress leads to social behavioral deficits in pre-weaned rodents, and later depressive-like symptoms in adolescent rodents (Raineki et al., 2012). As for the morphological substrate of these behavioral changes, we have previously shown that MD decreased numbers of NeuN-expressing neurons and parvalbumin-expressing interneurons in the prefrontal cortex (Aksić et al., 2014, 2021), as well as the decreased overall number of NeuN-expressing neurons in the amygdala and nucleus accumbens (Aleksić et al., 2016).

Psychiatric patients often show aberrant brain activity in regions implicated in emotion and reward processing, such as the amygdala and nucleus accumbens (Pankow et al., 2013). The amygdala is a limbic structure deep within the temporal lobe that is involved in processing emotions and regulating behavioral and physiological responses to stressors (Kennedy et al., 2007; Bryant et al., 2008). Amygdala hyperactivity has been observed in several functional neuroimaging studies investigating anxiety disorders (Xie et al., 2021). Together with the hypothalamus and ventral striatum, the amygdala is implicated in mood disorders, including depression and anxiety, and in substance abuse (Medina et al., 2023). Dysfunction in the corticolimbic circuitry also predicts increased fear responses, hypersensitivity to stress and negative behavioral outcomes (Guadagno et al., 2021). One of the main parts of the ventral striatum, as a part of limbic system, is the nucleus accumbens, a region with the capacity to mediate a diverse range of stress responses by interfacing limbic, cognitive and motor circuitry (Lemos et al., 2012). The stress-associated changes in limbic plasticity, dopamin release in the ventral tegmental area and reward—related behaviors point to the nucleus accumbens as a brain region particularly sensitive to stress (Perrotti et al., 2004).

The disruption of inhibitory circuits may underlie some of the clinical features in various psychiatric disorders (Schmalbach et al., 2015; Aksić et al., 2021). The GABAergic neurons play a fundamental role in the proper maturation of neural circuitry during postnatal development (Ben-Ari et al., 2004; Marguet et al., 2015). Calcium-binding proteins are important in the defense of neurons against excitotoxic damage, particularly for immature neurons due to their sensitivity to the influx of Ca2+ ions (Hogan and Berman, 1993). GABAergic interneurons can be defined further by the presence of one of three calcium binding proteins: parvalbumin (PV), calbindin (CB), or calretinin (CR). Only 20% of neurons in the amygdala are GABAergic interneurons but they have considerable role in the control of excitatory principal neurons (Hajos, 2021). In the amygdala, CB—immunopositive (CB+) and PV+ interneurons are localized primarily in the basolateral nucleus, as well as in the cortical amygdalar group of nuclei, while their number in the medial nucleus of the amygdala is very small (McDonald and Betette, 2001). CB+ and PV+ are most often colocalized on the same interneuron in the amygdaloid complex of nuclei. PV+ interneurons in the amygdala are basket and candle cells, similar to the same cells in the cerebral cortex and make up 50% of inhibitory interneurons (McDonald and Betette, 2001). In the nucleus accumbens approximately 95% of the neurons are GABAergic medium spiny neurons (Robison and Nestler, 2011) while the remaining interneurons express somatostatin (SST), parvalbumin, or the calcium-binding protein calretinin and calbindin. There are not many studies dealing with GABAergic inhibitory interneurons in the nucleus accumbens and the data are non-consistent. The PV+ interneurons in the nucleus accumbens are multipolar neurons, medium in size with greater density in rostral pole. Their density is lower than in the dorsal striatum, but with no difference in the distribution between shell and core (Totterdell and Meredith, 1997). The CB+ interneurons are more densely populated in the core of the nucleus accumbens compared to its shell (Tan et al., 1999). CR+ interneurons synapse predominantly on the dendritic shafts of other GABA interneurons, thus may act to disinhibit pyramidal neurons (Meskenaite, 1997; Gonchar and Burkhalter, 1999). CR+ interneurons account for 25% of all GABA-ergic interneurons in the basolateral amygdala (Hajos, 2021). The distribution of CR+ interneurons in the nucleus accumbens is higher than in dorsal striatum. These interneurons are uni-or bipolar cells which distribution is the same in the core and shell of the nucleus accumbens, but there is no consensus whether their density changes from the rostral to the caudal pole and from the medial to the lateral part of the nucleus (Hussain et al., 1996). Also, there is no clear evidence that PV and CR colocalize in the same interneuron (Hussain et al., 1996).

**Figure 1:**
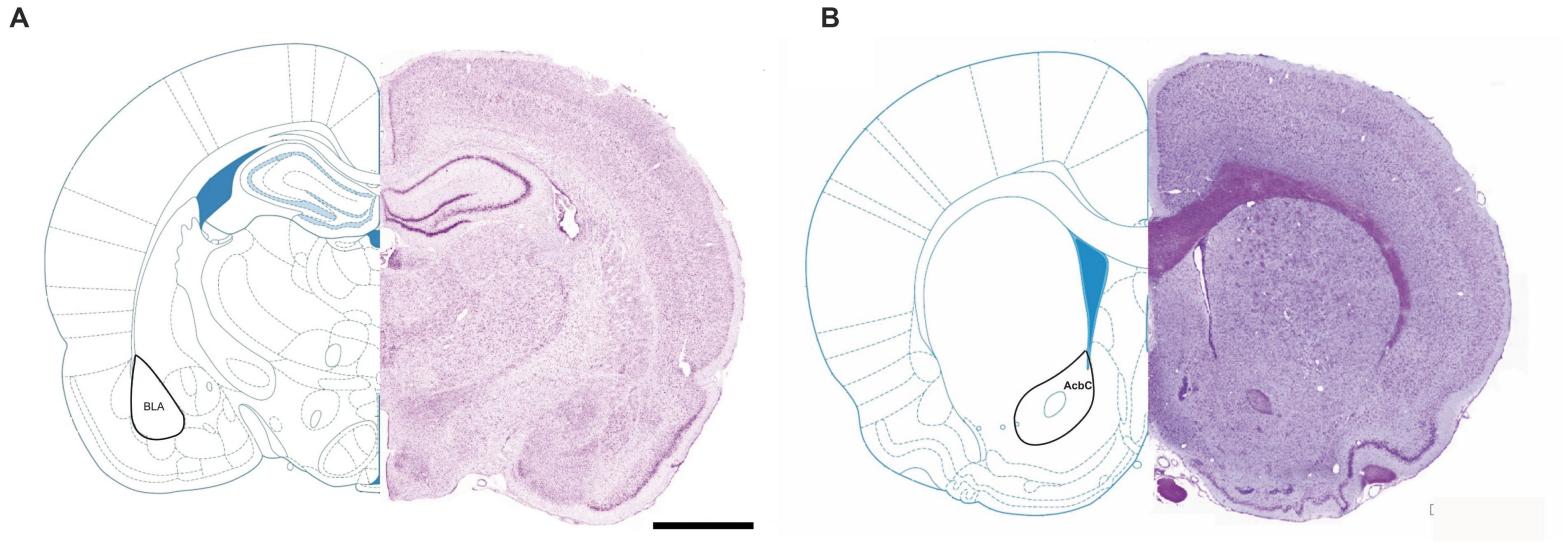
Representative micrographs of Nissl-stained rat brain sections, with labeled nucleus accumbens core **(A)** and basolateral amygdala **(B)**. AcbC, nc. accumbens core; BLA, basolateral amygdala.

The aim of this study was to investigate the long-term effects of MD on the number and size of GABAergic interneurons which express parvalbumin, calbindin and calretinin in the rat basolateral amygdala (BLA) and the nucleus accumbens core (AcbC) (Figure 1).

## Materials and methods

### Animals and procedures

A male and four nulliparous female 3-month-old Wistar rats were put together in a standard plexiglass cage, in a temperature controlled room. The lights were on from 07:00 a.m. to 07:00 p.m., and water and food were available *ad libitum*. Two weeks later, as the dams got pregnant, the males were removed and the dams were checked twice daily for delivery. The day of delivery was denoted as the postnatal day zero (P0). On P9, the litters were weight and then subjected to the MD procedure, as published previously (Ellenbroek et al., 1998; Roceri et al., 2002). The dams were removed from the litter at 10:00 a.m., and the pups were returned to their home cage afterwards. The pups remained in their home cage at room temperature for 24 h. On P10, the pups were weighed again, and the dams were returned to their cages. As a control experiment, the dams of were briefly (3 min) removed from their home cages and the pups were weighed on both P9 and P10. The litters were further left undisturbed except for the routine cleaning until P21, when they were weaned and assigned to new cages according to the sex. To avoid sexual dimorphism, only male rats were used for morphological studies (Woolley and McEwen, 1992) as was the case with many previous studies (Own and Patel, 2013; Vivinetto et al., 2013). The animals were sacrificed at P60, as young adults. All experiments were carried out according to the NIH Guide for Care and Use of Laboratory Animals, and were approved by the Local Bioethics Committee 323-07-10153/2016-05/3.

### Tissue processing and immunofluorescece staining

For morphological analysis, the animals from control and MD groups were anaesthetized with chloral hydrate (3 mg/kg, i.p.) and transcardially perfused with fixative (4% formaldehyde in 0.1 M phosphate buffer solution), at 60 days of age. The brains were post-fixed for 24 h at +4°C and cryoprotected by infiltration with sucrose for 2 days at 4°C (20% sucrose in 0.1 M phosphate buffer). Brains were frozen by immersion in 2-methyl-butane (Fluka) precooled to −80°C and then stored at −80°C. On a cryostat (Leica Instruments, Nußloch Germany) spaced-serial frontal sections were cut at 25-μm thickness. The sections were collected in a standard sequence so that four sections 250 μm apart were present on each slide, using SuperFrost Plus glass slides (Menzel Braunschweig, Germany).

Immunofluorescence staining was performed after antigen retrieval (0.01 M sodium citrate solution, pH 9.0, for 30 min at 80°C in a water bath). To block the nonspecific binding of the secondary antibody, sections were rinsed in a 5% normal serum from the species in which the secondary antibody was produced, diluted in 0.1 M phosphate-buffered saline (PBS, pH 7.3), and supplemented with 0.2% Triton X-100 and 0.02% sodium azide for 1 h at room temperature (RT). The primary antibodies (anti-mouse parvalbumin, 1:1000, Sigma; anti-rabbit calretinin, 1:1000, Sigma; or anti-calbindin 1:1000, Sigma) were diluted in PBS (pH 7.3) containing 0.5% lambda-carrageenan (Sigma) and 0.2% sodium azide and applied to the sections for 2 days at 4°C. After several washes in PBS, the sections were incubated for 2 h at RT with the appropriate Cy3-conjugated secondary antibodies diluted at 1:200 in PBS containing 0.5% lambdacarrageenan and 0.2% sodium azide. Following a subsequent wash in PBS, the nuclear counterstaining was performed with bis-benzimide solution (Hoechst dye 33258, 5 μg/mL in PBS, Sigma) 10 min at RT. Slices were washed in PBS, covered by a coverslip, and allowed to dry for 24 h before analysis. Specificity of staining was controlled by replacing the primary antibody with the normal serum from the animal in which the antibody was produced, which resulted in the absence of fluorescent signal.

### Cresyl-violet staining

In order to visualize brain structures, every 10th microscope slide (containing 4 sections at 250-μm distance from each other), was stained using standard cresyl-violet staining protocol, as previously described (Jakovljević et al., 2022). Briefly, the sections were rinsed for 3 min in 2 changes of xylene, 95, and 70% ethanol and distilled water. The sections were incubated in cresyl violet dye (Sigma) for 10 min at 60°C, rinsed in distilled water and incubated in a series of ethanols with concentration gradient 70, 95, 100%, 3 min each, and finally cleared in xylene for 5 min, prior to drying and applying Entellan mounting medium (Sigma).

### Image acquisition and quantitative analysis of immunolabeled neurons

Quantitative analyses were performed similar as previously described (Aleksić et al., 2016). Spaced-serial sections (250 μm apart) were used for quantifications. The borders of the amygdala and nucleus accumbens regions were defined by the nuclear staining pattern using ×10 objective. The profile density of various subclasses of interneurons was estimated by counting the immunolabeled cells within the delineated regions of interest.

Images for the cell body area were taken on the fluorescent confocal microscope (Zeiss LSM 510) with a 40× objective and analyzed in Image J software (Adobe, San Jose, CA), using an 1-cm grid. The cell body area of immunolabeled interneurons was estimated in spaced serial sections of the rat brains at the same distance from the bregma (−2.52 mm for the nucleus accumbens and −2.76 mm for the amygdala) and was expressed per unit area (mm^2^), which will further be referred to as the “cell soma area.” At least 200 random microscope fields (area of 400 μm^2^) were counted bilaterally in the nucleus accumbens and the amygdala of each section.

Left and right sides were evaluated in four sections each. All results shown are averaged bilateral values. The counts were performed on coded microscope slides by one observer.

### Statistical analysis

All numerical data are presented as group mean values with standard deviations (SD). Morphological analysis was performed for the left and right side separately, and as no difference between the sides was observed, data were pooled together. After the Shapiro–Wilk test confirmed normal distribution, the comparisons between groups were performed using Student’s *t* test for two independent samples, with the threshold value for acceptance of the difference set at 5%.

## Results

There was no significant weight loss neither in the MD group between P9 and P10 (15.3 g vs. 14.7 g on P9 and P10, respectively, *p* = 0,64, *n* = 2 litters or at least 8 pups each), nor between MD and control group on P10 (16.1 vs. 15.4 for CON and MD, respectively, *p* = 0,77, *n* = 2 litters or at least 8 pups each). Therefore, stress from MD protocol did not significantly affect the body weight of rat pups.

### Maternal deprivation decreases the number of GABAergic interneurons in the basolateral amygdala

**Figure 2:**
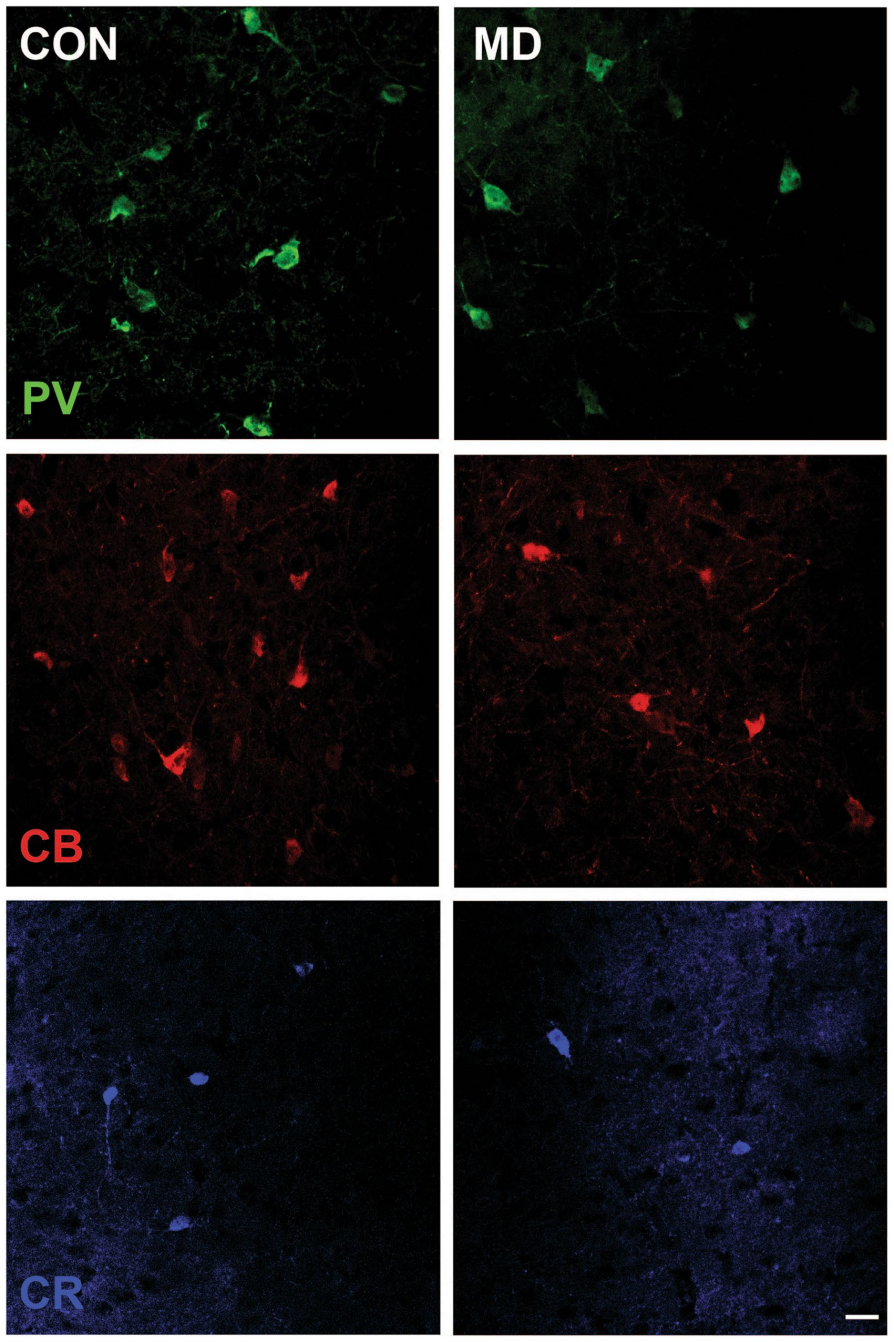
Representative micrographs of the PV^+^, CB^+^, and CR^+^ neurons in the BLA of control (CON, left panels) and maternally deprived rats (MD, right panels). Inset represents the schematic drawing of the investigated area. Scale bar: 25 μm.

**Figure 3:**
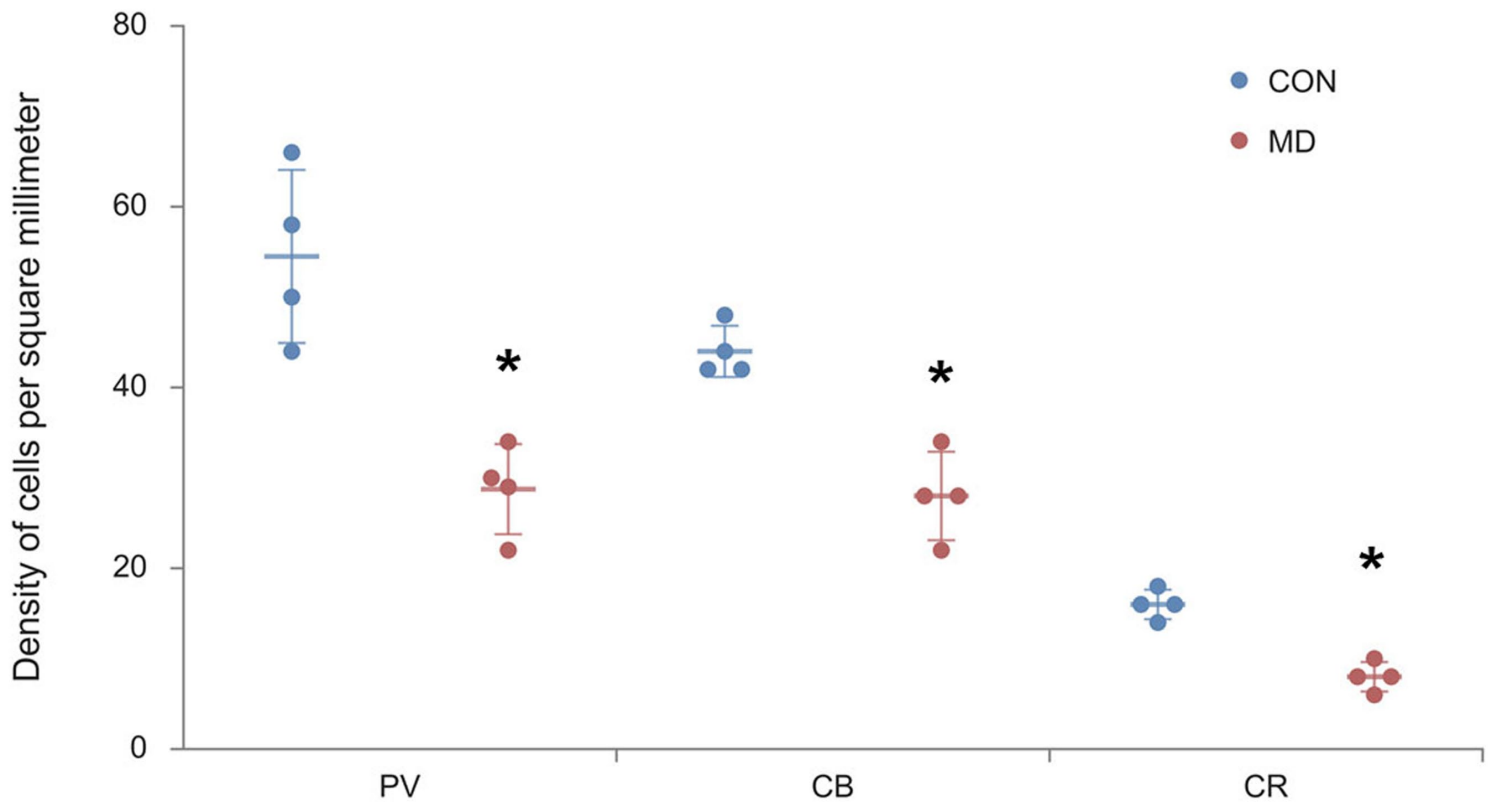
Profile densities of PV^+^, CB^+^, and CR^+^ neurons in the BLA. Results are presented as the mean values ± SD. The asterisks indicate significant differences between group mean values (two-tailed *t* test, *p* < 0.05).

Profile densities (number of cells per surface area) of GABAergic interneurons in sections of the rat brain were counted according to the distance from the bregma (Figure 1). We immunostained the control and MD rat brain sections for PV, CB, and CR (Figure 2). In the BLA, the profile density of the PV^+^ interneurons was 54.5 ± 9.5 cell/mm^2^ vs. 28.7 ± 4.9 cell/mm^2^ for the control group vs. MD, respectively [*t*(6) = 4.77; *p* = 0.003]. The profile density of the CB^+^ interneurons was 44 ± 1.4 cell/mm^2^ vs. 28 ± 2.9 cell/mm^2^ for the control group vs. MD, respectively [*t*(6) = 5.657; *p* = 0.001]. The profile density of the CR^+^ interneurons was 16 ± 1.5 cell/mm^2^ vs. 8 ± 1.7 cell/mm^2^, for the control group vs. MD, respectively [*t*(6) = 6.928; *p* < 0.001]. These differences were statistically significant, *p* = 0.003, *p* = 0.001, and *p* = 0.0004, for PV, CB, and CR, respectively (Figure 3). We conclude that maternal deprivation reduces numbers of calcium-binding protein expressing interneurons in the amygdala.

### Maternal deprivation decreases the number of GABAergic interneurons in the nucleus accumbens core

**Figure 4:**
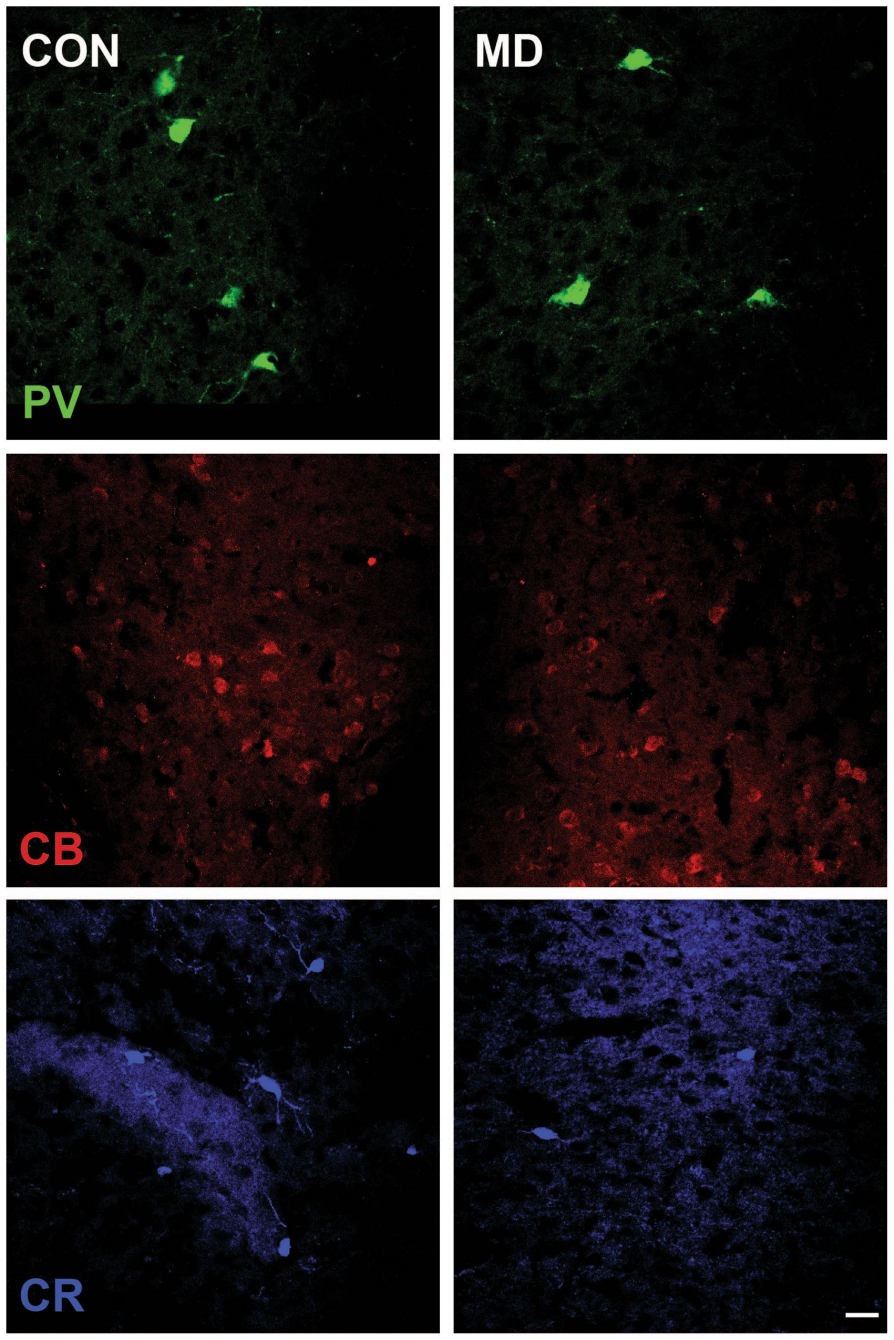
Representative micrographs of the PV^+^, CB^+^, and CR^+^ neurons in the AcbC of control (CON, left panels) and maternally deprived rats (MD, right panels). Inset represents the shematic drawing of the investigated area. Scale bar: 25 μm.

**Figure 5:**
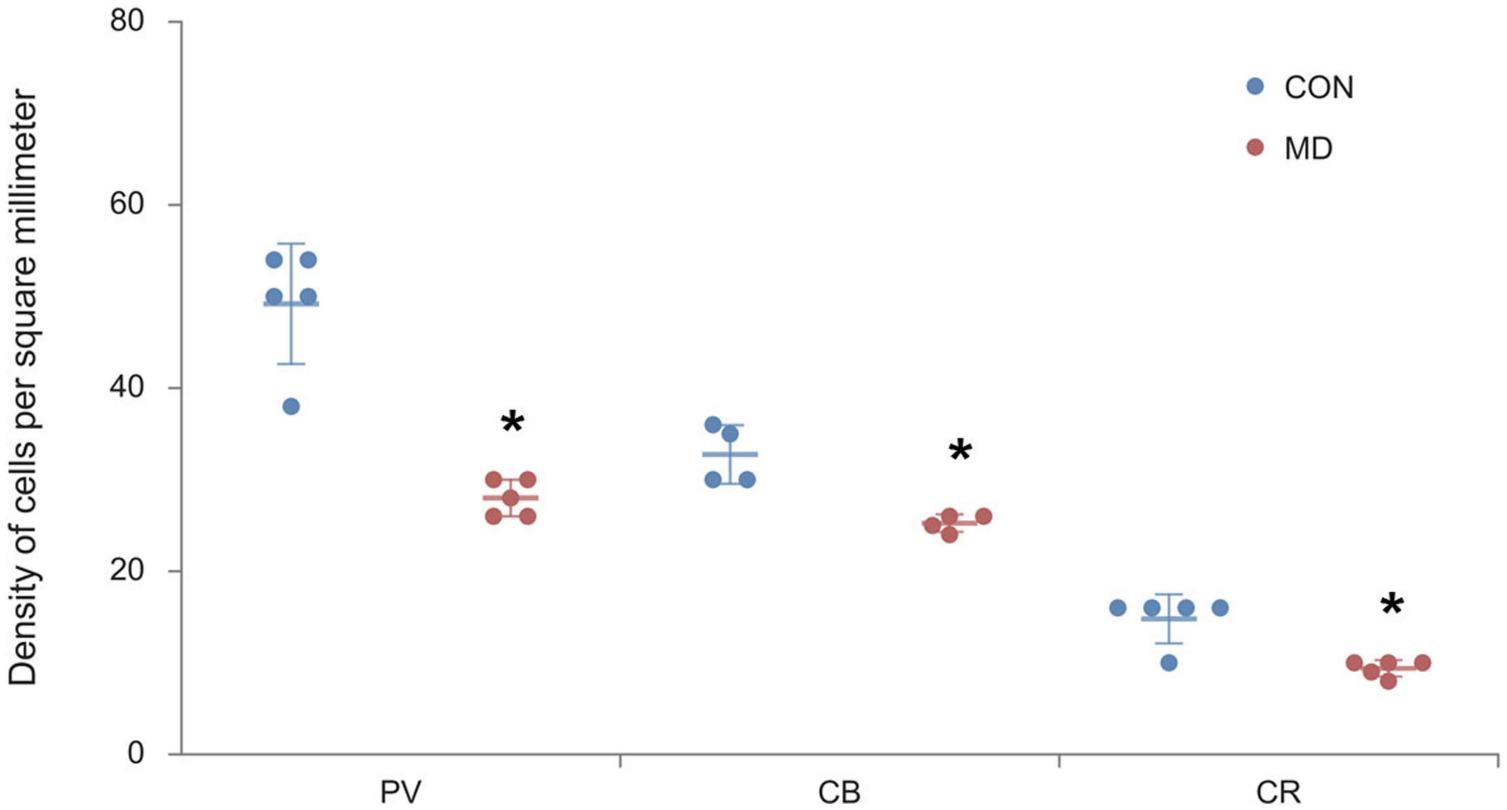
Profile densities of PV^+^, CB^+^, and CR^+^ neurons in the AcbC. Results are presented as the mean values ± SD. The asterisks indicate significant differences between group mean values (two-tailed *t* test, *p* < 0.05).

We immunostained the control and MD rat brain sections for PV, CB, and CR (Figure 4) containing AcbC. The profile density of the PV^+^ interneurons was 49 ± 2.9 cell/mm^2^ vs. 28 ± 0.9 cell/mm^2^ for the control group vs. MD, respectively [*t*(8) = 6.9; *p* < 0.001]. The profile density of the CB^+^ interneurons was 32.75 ± 3.2 cell/mm^2^ vs. 25.25 ± 0.9 cell/mm^2^ for the control group vs. MD, respectively [*t*(8) = 4.48; *p* = 0.004]. The profile density of the CR^+^ interneurons was 14.8 ± 2.7 cell/mm^2^ vs. 9.48 ± 0.8 cell/mm^2^, for the control group vs. MD, respectively [*t*(8) = 4.21; *p* = 0.003]. These differences were statistically significant, *p* = 0.0001, *p* = 0.004, and *p* = 0.003, vor PV, CB, and CR, respectively (Figure 5). We conclude that maternal deprivation reduces numbers of calcium-binding protein expressing interneurons in the nucleus accumbens as well.

### The effect of maternal deprivation on the reduction of cell body areas of GABAergic interneurons in the AcbC and BLA

**Figure 6:**
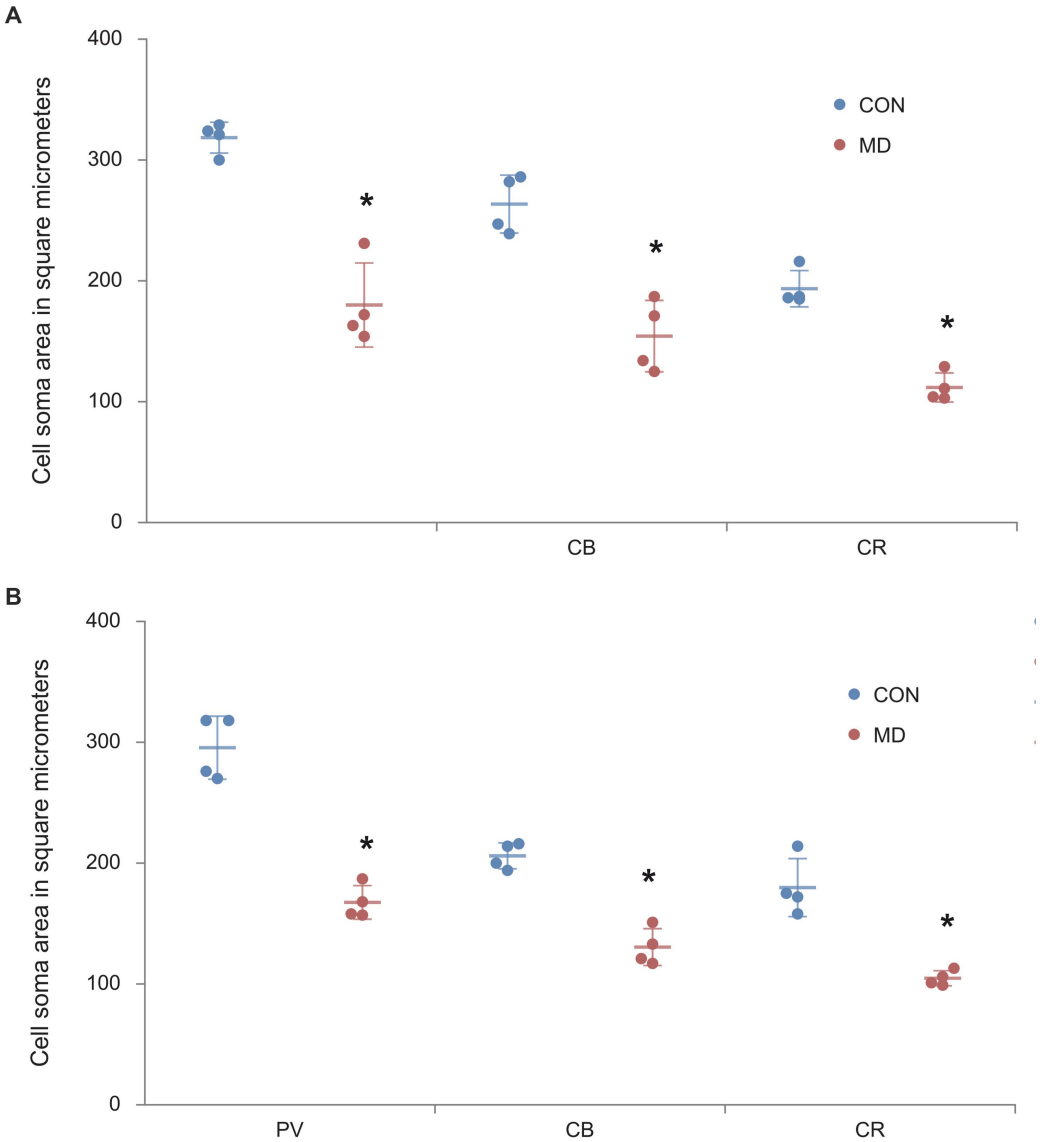
The cell body areas of the PV^+^, CB^+^, CR^+^ interneurons in the AcbC **(A)** and BLA **(B)**. Results are presented as the mean values ± SD. The asterisks indicate significant differences between group mean values (two-tailed *t* test, *p* < 0.05).

In our previous study we demonstrated that NeuN+ principal neurons in the amygdala and nucleus accumbens had smaller cell bodies after maternal deprivation, compared to controls (Aleksić et al., 2016). Thus, we also measured the cell body area of the GABAergic interneurons. The average cell body area of the PV^+^ interneurons in the AcbC of the control group was 287.8 ± 15.1 μm^2^, while in the MD group it was 161.06 ± 3.4 μm^2^ [*t*(6) = 7.473; *p* < 0.001]. The cell body area of the CB^+^ interneurons in the control group was 203.49 ± 6.5 μm^2^, while in the MD group it was 123.6 ± 4.8 μm^2^ [*t*(6) = 5.722; *p* = 0.001]. The cell soma area of the CR^+^ interneurons in the control group was 178 ± 12.1, while in the MD group it was 104.7 ± 3.2 μm^2^ [*t*(6) = 8.399; *p* < 0.0001]. These differences were statistically significant, *p* = 0.0002, *p* = 0.001, and *p* = 0.00001, vor PV, CB, and CR, respectively (Figure 6A).

As for the BLA, the cell body area of the PV^+^ interneurons in the amygdala of the control group was 316.7 ± 8.5 μm^2^, while in the MD group it was 185.9 ± 23.2 μm^2^ [*t*(6) = 8.36; *p* < 0.001]. The cell body area of the CB^+^ interneurons in the control group was 269.05 ± 15.2 μm^2^, while in the MD group it was 186.8 ± 30.5 μm^2^ [*t*(6) = 8.043; *p* < 0.001]. The cell body area of the CR^+^ interneurons in the control group was 184.08 ± 1.1 μm^2^, while in the MD group it was 103 ± 0.9 μm^2^ [*t*(6) = 6.021; *p* < 0.0001]. These differences were statistically significant, *p* = 0.0001, *p* = 0.0002, and *p* = 0.0009, for PV, CB, and CR, respectively (Figure 6B). We conclude that not only the number of interneurons, but also their cell body size is reduced after MD.

## Discussion

In this study, we investigated the long term effects of maternal deprivation on the GABAergic interneurons in the nucleus accumbens and the amygdala. Our results have shown reduced numbers of PV^+^, CB^+^, and CR^+^ cells in both, the nucleus accumbens and the amygdala of the maternally deprived rats, which has also been accompanied by a reduction in size of the neuron cell body.

Alterations in the expression of calcium binding proteins in various forebrain areas have been linked to anxiety—related behaviors and depression in humans and rodents (Lupien et al., 2009; Maciag et al., 2010). Our results suggest a decreased GABAergic tone in the amygdala and nucleus accumbens. It is important to know that, many aspects of GABAergic transmission in the BLA mature at the end of the first postnatal month (Ehrlich et al., 2013). The GABAergic tone in the amygdala reduces emotional arousal in social interactions, thereby decreased numbers of interneurons are implicated in increased anxiety behavior (Smith et al., 2000). Under resting conditions, the amygdala is inhibited by the extensive GABAergic network and exhibits low neuronal firing (Sanders and Shekhar, 1995). By contrast, a disinhibited amygdala leads to heightened activation upon chronic stress (Quirk and Gehlert, 2003; Barr et al., 2004), resulting in the increased sensitivity of to the environmental cues and individual’s hypervigilance which persist even after long period of recovery. Experimental exposure to dexametason in rats also caused the decrease in the number of CR^+^ and CB^+^ cells in the amygdala, due to decreased proliferation, altered phenotype differentiation and migration (Zuloaga et al., 2012). The decreased inhibitory synaptic transmission in the amygdala results from the loss of GABAergic interneurons which is associated with increased anxiety—like behaviors (Truitt et al., 2009). Mounting evidence has demonstrated that amygdala is one of the primary targets of chronic stress (Vyas et al., 2006; Roozendaal et al., 2009). Contrary to our results, Giachino et al. (2007) reported that rats exposed to maternal separation during the first 2 weeks of life and those exposed to prolonged maternal separation, had increased density of PV+ in the lateral nucleus of amygdala, but not in the basal nucleus. They also showed that CR+ and CB+ neuronal densities did not change in any nucleus of the amygdala analyzed. Gildawie et al. (2020) found sex-, age-, and region-specific effects of early life adversity on PNN and PV+ maturation. His work reveals that maternal deprivation does not change the density of perineuronal network, but leads to an increase in the density of PV+ neurons in the male basolateral amygdala in adolescence period comparing to females. Like in our study, they showed that GABAergic and extracellular maturation corresponds with major changes in neurocircuitry and plasticity. New evidence showing that early life stress alters perineuronal nets that play important role stabilizing synaptic inputs to developing inhibitory interneurons (Guadagno et al., 2021; Jakovljević et al., 2022). Maternal deprivation, as a model of early life stress, also affects the physiology of the nucleus accumbens. Restrain stress elevates c-Fos protein expression in numerous reward-related brain regions, including the nucleus accumbens shell and core (Barr et al., 2004). Perrotti et al. (2004) found that chronic stress increased FosB expression throughout the nucleus accumbens. FosB has been shown to increase the expression of metabotropic glutamate receptor subtype 2 (GluR2) in the nucleus accumbens (Kelz et al., 1999). Nucleus accumbens contains levels of corticotropin—releasing factor (CRF) receptors that are comparable to those in the amygdala (Aguilera et al., 2004; Lim et al., 2005). Schizophrenia patients show a substantial loss of various subclasses interneurons in the limbic system (Swanson et al., 1983). The loss of these interneurons presumably creates disruption in the modulation of cortical inputs to the nucleus accumbens.

It is worth mentioning in this context, that in our MD model we evaluated the numbers of principal cells, as well as interneurons in several other relevant structures, such as the hippocampus, prefrontal, cingulate and infralimbic cortices. In all these structures, structure volume, number of principal neurons as well as interneurons were decreased (Aksić et al., 2014, 2021), and the evidence for the increased oxidative stress were found (Marković et al., 2017). Additionally, the number and structure of perineuronal nets around PV+, as well as PV-neurons were altered in the lymbic cortex, but not in the hippocampus (Jakovljević et al., 2022). These findings are accompanied by reduced cholinergic input to those structures, as well as overall loss of dopaminergic neurons in the substantia nigra and ventral tegmental area (Marković et al., 2014; Kapor et al., 2020). MRI studies of human nucleus accumbens show the reduction in the concentration of GABA observed following situational stress in people. Reduced GABA concentration in the nucleus accumbens might reflect a reduction in GABAergic function resulting in reduced inhibitory potential in the nucleus accumbens associated neural circuitry in humans (Strasser et al., 2019).

Research to date suggests that long-term effects of postnatal manipulations cause diverse changes in the neurobiological and neuroendocrinological systems. In the light of neurodevelopmental changes, the development of CB+ and PV+ interneurons in the amygdala is most intensive during the first two postnatal weeks (Berdel et al., 1997). Moreover, this is the period when significant morphological changes occur in the formation of the basolateral nucleus of the amygdala (Berdel et al., 1997), which makes the developing cells particularly sensitive to stress. The development of CB+ interneurons begins even earlier, and it is assumed that these interneurons are therefore important for cell migration and differentiation (Berdel and Moryś, 2000). Presumably, PV+ interneurons in the amygdala, play a role in formation and maturation of synapses. The final distribution of CB+ and PV+ interneurons is formed around the 14th postnatal day, so it is assumed that any stress situation during the period after the birth, like the separation from the mother, my cause developmental changes which can be manifested later in life through various neuropsychiatric diseases (Berdel et al., 1997). Hyperactivity of the hypothalamic—pituitary—adrenal (HPA) axis, induced by early life stress and followed by increased levels of corticotropin—releasing hormone (CRH) and corticosterone (Fuchs and Flügge, 1998; Swaab et al., 2005). Increased cortisol levels (Matthews and Fava, 2000) lead to neuronal cell death, because the limbic system is highly sensitive to endogenous glucocorticoids during brain development (Cottrell, 1972). Exposure of neonatal rats to high levels of glucocorticoids leads to a reduction in the brain weight (Russo-Neustadt, 2003), reduction of dendritic spine density, neuronal atrophy and changes of the neuronal morphology, which may explain why the cell soma area of the PV^+^, CB^+^, CR^+^ interneurons is reduced in our study in both examined structures. Glucocorticoids have the ability to bind to the GABA A receptor and have been found to change its activity (Helmeke et al., 2008). Hence, exposure to early postnatal stressors such as maternal deprivation has the potential to disrupt the typical developmental trajectory of the GABAergic system, thereby establishing a foundation for the emergence of mental disorders in later stages of life.

In our previous study, we demonstrated that maternal deprivation led to a reduction in the size of the amygdala and nucleus accumbens in young adult male rats, along with a decrease in the quantity and soma areas of NeuN-positive cells present within these regions (Aleksić et al., 2016). Considering that the NeuN protein is the marker of almost all neurons (Wolf et al., 1996), including interneurons, this study confirms that the volume of the amygdala and nucleus accumbens has probably reduced through decrease in the number and size of the calcium binding PV^+^, CB^+^, and CR^+^ interneurons. This study furthers our understanding how early life stress affects inhibitory neurons in the amygdala and nucleus accumbens. Additional investigations are necessary to evaluate the comprehensive impact of the combined loss of principal cells (Aleksić et al., 2016) and inhibitory interneurons (present study) on the connectivity within the limbic system.

## Data availability statement

The raw data supporting the conclusions of this article will be made available by the authors, without undue reservation.

## Ethics statement

The animal study was reviewed and approved by Local Bioethics Committee 323-07-10153/2016-05/3.

## Author contributions

DA, JP, GA, VD, MH, and MjA performed experiments, collected data, and interpreted data. GR, JP, MV, IJ, and MaA analyzed data and wrote the manuscript. All authors contributed to the article and approved the submitted version.

## Funding

This study was supported by grants III 41020 and 175058 from the Ministry for Science and Environmental Protection of the Republic of Serbia.

## Conflict of interest

The authors declare that the research was conducted in the absence of any commercial or financial relationships that could be construed as a potential conflict of interest.

## Publisher’s note

All claims expressed in this article are solely those of the authors and do not necessarily represent those of their affiliated organizations, or those of the publisher, the editors and the reviewers. Any product that may be evaluated in this article, or claim that may be made by its manufacturer, is not guaranteed or endorsed by the publisher.
